# Supplementary material for: Ultrashort pulse biphoton source in lithium niobate nanophotonics at 2 μm
Source: Nanophotonics. 2024 Jun 13;13(18):3535–44. doi: 10.1515/nanoph-2024-0054 (PMC11501393; doi:10.1515/nanoph-2024-0054)
Supplement: Supplementary file 1 — Supplementary Material Details [file j_nanoph-2024-0054_suppl_001.pdf]

Supplementary material for Ultra-short Pulse Biphoton Source in Lithium Niobate Nanophotonics at 2 µm

James Williams, Rajveer Nehra, Elina Sendonaris,Luis Ledezma, Robert M. Gray, Ryoto Sekine, and Alireza Marandi

May 26, 2024

1 Multimode Light Generation

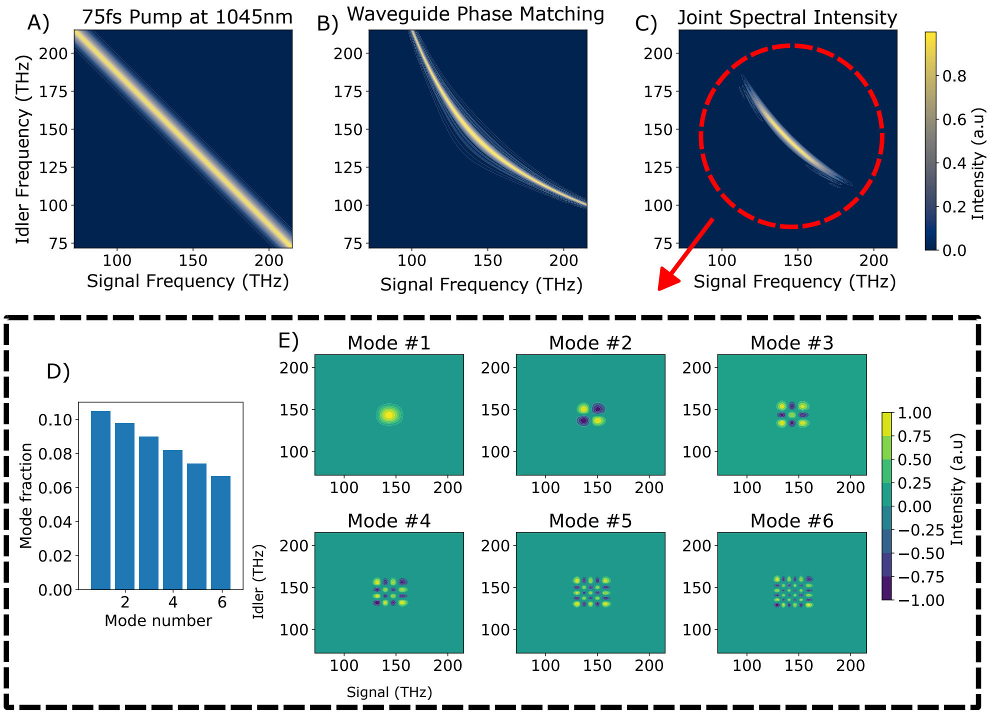

Figure 1: A) Energy conservation of the 75-fs pump used for our source. B) Phase matching (i.e momentum conservation) provided by our waveguide design. C) The joint spectral intensity, or the product of A) and B). D) A plot of the mode occupancy vs mode number for the first 6 spectro-temporal modes which comprise our JSI in C). E) Plots of the first 6 modes in the frequency domain.

As discussed in the main text, the light generated in our type-0 SPDC process is highly spectro-temporally multimode. These modes can be described as a set of time-dependent electric fields, all of which are orthogonal to each other, thereby forming a complete mode basis [1]. The modes themselves can be found by performing a Bloch-Messiah decomposition on the joint-spectral intensity function (JSI) of the signal and idler photons [2]. Fig.1 shows the energy conservation of the pump, the phase matching function calculated from the dispersion of the waveguide, and their product, the JSI. The occupancy of the first 6 modes are plotted in Fig.1D and their structure in the frequency domain is plotted in Fig.1E. The shape of the modes is determined both by the shape of the JSI

and the frequency space upon which it is measured.

Because our JSI cannot be decomposed into the product of two independent frequency distributions, it is inherently composed of multiple entangled modes. These modes pose a problem when interfering multiple independent sources of photons together to create a larger quantum state of interest. Interference acts as a measurement of the relative mode of the two incoming photons. If both photons share the same mode, then they interfere strongly, whereas photons in different modes experience no interference. The visibility of the interference between two independent but identical sources of biphotons is limited to approximately $\frac{1}{N}$ where N is the number of modes present [3].

The number of modes in emitted signal and idler pairs can be reduced by engineering the dispersion of the waveguide to meet certain group velocity mismatch requirements [4, 5], or by engineering the poling domains to remove fringes caused by the $sinc^2$ shape of the phase matching function provided by periodic poling [6, 7]. For degenerate SPDC in the type-0 configuration, reducing the mode number is challenging as the phase matching function runs parallel to the energy conservation of the pump near degeneracy, thereby causing the JSI to take on a narrow and inseparable elliptical shape. Filtering can also be employed to reduce the mode number as this limits measurements to a local region of the JSI. If the JSI is relatively constant in this region, then the source will appear to be single mode. This does however come at the cost of a reduced rate, as well as the introduction of a distinguishable thermal component into the state as a result of the presence of vacuum at one of the input ports of the filter [2].

2 Determining Losses via Parametric Generation

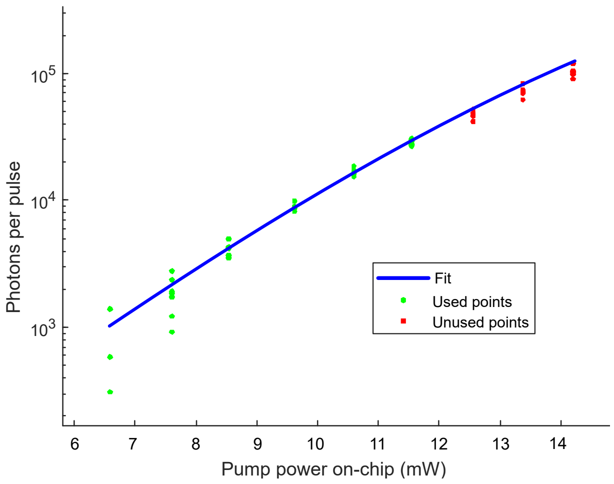

Figure 2: A plot of the average number of photons per pulse vs input pump power.

In order to accurately calculate the pair generation rate, we must know the pump power on-chip. We can measure the pump power off-chip before the reflective objective, and then infer the on-chip pump power by multiplying by the input loss. While the input loss cannot be directly measured, we can calculate it by measuring the throughput loss and subtracting the output loss to find the input loss. The throughput loss is measured by sending in 1 µW of pump power to avoid depletion effects, and then measuring the received power at the output. The output loss is calculated using the same methods presented in [8]. The output average photon number is first measured verses the input pump power. This curve is then fitted with the function $N_{avg} = \eta sinh(\sqrt{\beta P})^2$ where η is the output loss and β is the nonlinear gain. Knowing the exact input loss is not necessary to compute η as changing the input loss (i.e changing the scale of the x-axis) will only affect the calculated value of β. After calculating the input loss, the curve can be fitted again with the correct x-axis to determine β.

Fig.2 shows the fitted photon number data. We extract a loss of 9 dB by fitting the raw data from 5 separate

Reference	Signal//Idler Wavelength	Platform	CAR	Pair Rate Hz/mW	Pair Rate $Hz/mW/GHz$	Pair Rate $Hz/mW/GHz/cm^2$	Heralded $g_H^{(2)}(0)$
[9]	1570nm/1570nm	5mm TFLN Waveguide	67000 ± 714	x	460 $\cdot 10^3$	1.84 $\cdot 10^6$	0.022±0.004
[10]	1500nm/1500nm	6mm TFLN Waveguide	599	279$\cdot 10^9$	11.5 $\cdot 10^6$	31.9 $\cdot 10^6$	x
[11]	1312nm/1557nm	40mm TFLN Waveguide	270000	15 $\cdot 10^9$	3.1 $\cdot 10^6$	19 $\cdot 10^3$	0.001
[12]	1530nm/1570nm	300μm TFLN Waveguide	6900 ± 200	x	28.6±0.54 $\cdot 10^3$	3.18±0.06 $\cdot 10^6$	x
[13]	1531nm/1571nm	5mm TFLN Waveguide	152710±32772	13$\cdot 10^9$	324±25 $\cdot 10^3$	1.37±0.01 $\cdot 10^6$	x
[14]	1578nm/1578nm	Large PPLN Waveguide	8000	36 $\cdot 10^9$	1.43 $\cdot 10^6$	229 $\cdot 10^3$	x
[15]	1550nm/1550nm	Large PPLN Waveguide	x	1.2 $\cdot 10^6$	160	17	x
[16]	1550nm/1550nm	Large PPLN Waveguide	x	14.4 $\cdot 10^6$	1.9 $\cdot 10^3$	213	x
[17]	1550nm/1550nm	AlN Ring Resonator	560	20$\cdot 10^6$	5.3 $\cdot 10^6$	x	0.088 ± 0.004
[18]	1550nm/1550nm	Bulk LBO	x	7.5 $\cdot 10^3$	x	x	x
[19]	1550nm/1550nm	InGaP Ring Resonator	1.4 $\cdot 10^4$	27.9 $\cdot 10^9$	x	x	x
[20]	1572nm/1542nm	AlGaAs Ring Resonator	2697 ± 260	20 $\cdot 10^9$*	200 $\cdot 10^9$*	x	0.004 ± 0.01
[21]	2100nm/2100nm	1mm Bulk PPLN	180 ± 50	320$\cdot 10^3$	x	x	x
[22]	1259nm/2015nm	Silicon-On-Insulator Waveguide	40.9 ± 9	5.9 $\cdot 10^6$*	x	x	0.23 ± 0.08
[23]	1289nm/2000nm	Silicon-on-Insulator Waveguide	114±4	5.7 $\cdot 10^3$†*	x	x	x
[24]	700nm/6300nm	Bulk $AgGaS_2$	706	14.6 $\cdot 10^6$	x	x	x
[25]	2000nm/2150nm	Silicon-on-Insulator Waveguide	25.7 ± 1.1	x	x	x	x
[26]	2080nm/2080nm	1mm Bulk PPLN	607 ± 185	98.33 $\cdot 10^3$	x	x	x
Our Work	2090nm/2090nm	5mm TFLN Waveguide	945±475	8.8±2.3 $\cdot 10^9$	440±115 $\cdot 10^3$	1.76±0.46 $\cdot 10^6$	0.027 ± 0.003

Table 1: x = did not report. † = estimated based on reported data. * = units of per mW^2

measurements. While this measurement gives the losses at 2 µm, we know from fiber-in fiber-out chip coupling measurements that the loss at 1 µm is approximately 1 dB more than that for 2 µm. Hence we conclude that the fiber coupling loss at 1 µm is 10 dB. Subtracting this from our throughput loss of 20 dB gives us 10 dB of input loss. Near the end of the curve, we have marked points in red which were not used in the fit. In the limit of large pump power, the assumptions underlying $N_{avg} = \eta sinh(\sqrt{\beta P})^2$ are violated, and the depletion of the pump pulse causes the experimental data to deviate from the model.

3 Detailed Comparison Table

Here we provide a more detailed comparison table which we reference in the main text. This table also includes $\chi^{(3)}$ sources which, while the constitute the majority of available mid-IR sources, are absent from the comparison plot as their rate scales as the square of the pump power instead of linearly as with $\chi^{(2)}$ sources.

References

[1] Benjamin Brecht, Dileep V Reddy, Christine Silberhorn, et al. "Photon temporal modes: a complete framework for quantum information science". In: *Physical Review X* 5.4 (2015), p. 041017.

[2] Martin Houde and Nicolás Quesada. "Waveguided sources of consistent, single-temporal-mode squeezed light: The good, the bad, and the ugly". In: *AVS Quantum Science* 5.1 (2023).

[3] Christopher L Morrison, Francesco Graffitti, Peter Barrow, et al. "Frequency-bin entanglement from domain-engineered down-conversion". In: *APL Photonics* 7.6 (2022).

[4] Warren P Grice, Alfred B U'Ren, and Ian A Walmsley. "Eliminating frequency and space-time correlations in multiphoton states". In: *Physical Review A* 64.6 (2001), p. 063815.

[5] Alfred B U'Ren, Christine Silberhorn, Reinhard Erdmann, et al. "Generation of pure-state single-photon wavepackets by conditional preparation based on spontaneous parametric downconversion". In: *arXiv preprint quant-ph/0611019* (2006).

[6] P Ben Dixon, Jeffrey H Shapiro, and Franco NC Wong. "Spectral engineering by Gaussian phase-matching for quantum photonics". In: *Optics express* 21.5 (2013), pp. 5879–5890.

[7] CJ Xin, Jatadhari Mishra, Changchen Chen, et al. "Spectrally separable photon-pair generation in dispersion engineered thin-film lithium niobate". In: *Optics Letters* 47.11 (2022), pp. 2830–2833.

[8] Luis Ledezma, Ryoto Sekine, Qiushi Guo, et al. "Intense optical parametric amplification in dispersion-engineered nanophotonic lithium niobate waveguides". In: *Optica* 9.3 (2022), pp. 303–308.

[9] Jie Zhao, Chaoxuan Ma, Michael Rüsing, et al. "High quality entangled photon pair generation in periodically poled thin-film lithium niobate waveguides". In: *Physical review letters* 124.16 (2020), p. 163603.

[10] Guang-Tai Xue, Yun-Fei Niu, Xiaoyue Liu, et al. "Ultrabright multiplexed energy-time-entangled photon generation from lithium niobate on insulator chip". In: *Physical Review Applied* 15.6 (2021), p. 064059.

[11] Matthias Bock, Andreas Lenhard, Christopher Chunnilall, et al. "Highly efficient heralded single-photon source for telecom wavelengths based on a PPLN waveguide". In: *Optics express* 24.21 (2016), pp. 23992–24001.

[12] Bradley S Elkus, Kamal Abdelsalam, Ashutosh Rao, et al. "Generation of broadband correlated photon-pairs in short thin-film lithium-niobate waveguides". In: *Optics express* 27.26 (2019), pp. 38521–38531.

[13] Usman A Javid, Jingwei Ling, Jeremy Staffa, et al. "Ultrabroadband entangled photons on a nanophotonic chip". In: *Physical Review Letters* 127.18 (2021), p. 183601.

[14] Yuting Zhang, Hao Li, Tingting Ding, et al. "Scalable, fiber-compatible lithium-niobate-on-insulator micro-waveguides for efficient nonlinear photonics". In: *Optica* 10.6 (2023), pp. 688–693.

[15] A Yoshizawa, R Kaji, and H Tsuchida. "Generation of polarisation-entangled photon pairs at 1550 nm using two PPLN waveguides". In: *Electronics Letters* 39.7 (2003), p. 1.

[16] Shigehiko Mori, Jonas Söderholm, Naoto Namekata, et al. "On the distribution of 1550-nm photon pairs efficiently generated using a periodically poled lithium niobate waveguide". In: *Optics communications* 264.1 (2006), pp. 156–162.

[17] Xiang Guo, Chang-ling Zou, Carsten Schuck, et al. "Parametric down-conversion photon-pair source on a nanophotonic chip". In: *Light: Science & Applications* 6.5 (2017), e16249–e16249.

[18] Tae-Gon Noh, Heonoh Kim, Chun Ju Youn, et al. "Noncollinear correlated photon pair source in the 1550 nm telecommunication band". In: *Optics express* 14.7 (2006), pp. 2805–2810.

[19] Mengdi Zhao and Kejie Fang. "InGaP quantum nanophotonic integrated circuits with 1.5% nonlinearity-to-loss ratio". In: *Optica* 9.2 (2022), pp. 258–263.

[20] Trevor J Steiner, Joshua E Castro, Lin Chang, et al. "Ultrabright entangled-photon-pair generation from an Al Ga As-On-insulator microring resonator". In: *PRX Quantum* 2.1 (2021), p. 010337.

[21] Shashi Prabhakar, Taylor Shields, Adetunmise C Dada, et al. "Two-photon quantum interference and entanglement at 2.1 μm". In: *Science advances* 6.13 (2020), eaay5195.

[22] S Signorini, M Sanna, S Piccione, et al. "A silicon source of heralded single photons at 2 μ m". In: *APL Photonics* 6.12 (2021), p. 126103.

[23] M Sanna, D Rizzotti, S Signorini, et al. "An integrated entangled photons source for mid-infrared ghost spectroscopy". In: *Quantum Sensing and Nano Electronics and Photonics XVIII*. Vol. 12009. SPIE. 2022, pp. 143–151.

[24] Mohit Kumar, Pawan Kumar, Andres Vega, et al. "Mid-infrared photon pair generation in AgGaS2". In: *Applied Physics Letters* 119.24 (2021), p. 244001.

[25] Lawrence M Rosenfeld, Dominic A Sulway, Gary F Sinclair, et al. "Mid-infrared quantum optics in silicon". In: *Optics Express* 28.25 (2020), pp. 37092–37102.

[26] Adetunmise C Dada, Jedrzej Kaniewski, Corin Gawith, et al. "Near-Maximal Two-Photon Entanglement for Optical Quantum Communication at 2.1 μ m". In: *Physical Review Applied* 16.5 (2021), p. L051005.
